# Clinicopathological and Radiological Profile of Gallbladder Carcinoma: A Cross-Sectional Study From a Tertiary Care Center in Tripura, India

**DOI:** 10.7759/cureus.104696

**Published:** 2026-03-05

**Authors:** Gowtham K Gudimetla, Krishna Chakma, Suryadipta Ghosh, Sambit Debbarman, Diptendu Chaudhuri, Abantika Nath, Annu Priya

**Affiliations:** 1 General Surgery, Agartala Government Medical College and Govind Ballabh Pant Hospital, Agartala, IND

**Keywords:** american joint committee on cancer (ajcc), gallbladder carcinoma, gallbladder histopathology, northeast india, radiological findings

## Abstract

Background

Gallbladder carcinoma is an aggressive malignancy with marked geographic variation in incidence within India, particularly affecting the northern and northeastern regions. Given the limited evidence from Tripura, this study aimed to describe the clinicopathological and radiological profile of gallbladder carcinoma patients presenting to a tertiary care center in Tripura, India.

Methods

A prospective, hospital-based, cross-sectional study was conducted in the Department of General Surgery, Agartala Government Medical College and Govind Ballabh Pant Hospital (AGMC & GBPH), Agartala, India, from May 2024 to November 2025. All patients with a confirmed diagnosis of gallbladder carcinoma during the study period were eligible. Of 123 diagnosed patients, 104 were included after applying the exclusion criteria. Data were collected using a structured proforma covering sociodemographic characteristics, clinical presentation, laboratory parameters, radiological findings, histopathology, and tumor staging. Staging was performed according to the American Joint Committee on Cancer (AJCC) 8th edition classification. Data were analyzed using IBM SPSS Statistics for Windows, Version 26 (Released 2018; IBM Corp., Armonk, NY, USA).

Results

The mean age of participants was 61.4 ± 8.8 years, with more than half of the participants (n = 59, 56.7%) aged over 60 years. A marked female predominance was observed (n = 92, 88.5%). Most patients belonged to lower socioeconomic strata (n = 52, 50.0%) and resided in rural areas (n = 78, 75%). Abdominal pain was the most common presenting symptom, while icterus was the most frequent clinical sign. Elevated carbohydrate antigen 19-9 (CA 19-9) was observed among 69 (66.3%) patients, and elevated carcinoembryonic antigen (CEA) levels were observed in 77 (74%) patients. On ultrasonography (USG), a gallbladder mass was detected in 82 (78.8%) cases, while contrast-enhanced computed tomography (CECT) identified mass lesions in 94 (90.4%) cases. Histopathologically, adenocarcinoma was the predominant subtype, with well-differentiated tumors accounting for 41 (39.4%) cases. Advanced disease at presentation was frequent, with T3-T4 tumors in 72 (69.2%) patients, nodal involvement in 67 (64.4%), and distant metastasis in 28 (26.9%) patients. Stage IVB was the most common AJCC stage (n = 42, 40.4%).

Conclusion

Gallbladder carcinoma patients in Tripura predominantly present with advanced-stage disease and exhibit a strong female preponderance. The high burden of locoregional and metastatic spread underscores the need for region-specific awareness, earlier diagnostic strategies, and strengthened referral pathways to improve outcomes in this high-risk population.

## Introduction

Carcinoma of the gallbladder is the most common malignancy of the biliary tract and represents one of the most aggressive gastrointestinal cancers, with an overall poor prognosis [1-3]. Early-stage disease is frequently asymptomatic and is often detected incidentally during cholecystectomy performed for presumed benign gallbladder disease. A large meta-analysis reported that the incidence of incidental gallbladder carcinoma following cholecystectomy averages approximately 0.6%, with individual studies demonstrating rates ranging up to 2.9% [4]. When present, clinical symptoms are typically vague and nonspecific, closely resembling those of cholelithiasis or chronic cholecystitis, which contributes to delayed diagnosis [2,5]. Approximately 90% of patients with gallbladder carcinoma have associated gallstones, highlighting the strong etiological role of cholelithiasis and chronic inflammation in gallbladder carcinogenesis [5].

Structural alterations associated with gallbladder carcinoma most commonly include a mass replacing the gallbladder lumen (reported in 40%-65% of cases), followed by focal or diffuse mural thickening (20%-30%) and intraluminal polypoidal lesions (15%-25%) [6]. On imaging, gallbladder carcinoma typically presents as a focal intraluminal polypoidal mass, focal or diffuse irregular thickening of the gallbladder wall, or a large mass lesion replacing the gallbladder lumen, reflecting the spectrum of macroscopic morphological patterns observed at presentation. Ultrasonography (USG) may demonstrate these appearances as intraluminal growths, asymmetric wall thickening, or a heterogeneous mass replacing the gallbladder. The tumor typically exhibits irregular or ill-defined margins, heterogeneous echotexture, and predominantly low echogenicity. On non-contrast computed tomography (CT), gallbladder carcinoma usually appears as a hypodense lesion, with irregular peripheral enhancement on the arterial phase following contrast administration [7,8].

Despite advances in imaging and diagnostic techniques, preoperative detection of early-stage gallbladder carcinoma remains challenging. Epidemiological data indicate that nearly four out of five patients with gallbladder carcinoma are diagnosed at an advanced or metastatic stage at the time of presentation [9]. Consequently, a substantial proportion of cases are identified only after the disease has progressed beyond the early, potentially curable stage, which largely accounts for the poor overall prognosis associated with this malignancy. Gallbladder carcinoma occurs two- to six-fold more frequently in women than in men, and its incidence increases with advancing age, peaking in the seventh and eighth decades of life. Emerging evidence also suggests a rising incidence among younger individuals [2,10,11]. The occurrence of gallbladder carcinoma closely parallels the prevalence of gallstone disease. Long-standing gallstones, particularly large calculi, are associated with a significantly increased risk, with studies reporting a four- to seven-fold elevation in risk [12].

According to recent estimates from the Global Cancer Observatory (GLOBOCAN), gallbladder cancer ranks 22nd in global cancer incidence and 20th in cancer-related mortality, with an estimated prevalence of 2.1 per 100,000 population [13]. In India, gallbladder cancer has an estimated prevalence of 2.6 per 100,000 population. It ranks 19th in overall cancer incidence and 15th among causes of cancer-related mortality, underscoring its relatively higher contribution to the national cancer burden compared to global patterns [14]. The burden of disease demonstrates substantial heterogeneity, with marked variation across gender, geographic regions, and ethnic groups [1,15]. While the incidence is relatively low in Western countries, India exhibits striking regional disparities. The burden is substantially higher in northern and north-eastern regions compared with southern India, where the disease remains relatively uncommon. Remarkably, gallbladder cancer is among the five most common gastrointestinal malignancies in the north-eastern states, with the highest reported incidence observed in the Kamrup district of Assam [11,12,15-17]. These marked geographic disparities highlight the importance of region-specific epidemiological and clinical investigations. Northeast India differs substantially from other regions of the country in terms of ethnicity, dietary practices, lifestyle patterns, and geographic characteristics, all of which may influence disease patterns. Despite this, published data on gallbladder carcinoma from the northeastern states remain limited, with particularly scarce evidence from Tripura. Against this background, the present study was undertaken with the aim of describing the clinicopathological and radiological profile of patients with gallbladder carcinoma diagnosed at a tertiary care center in Tripura, India.

## Materials and methods

Study design and setting

This was a prospective, observational, hospital-based cross-sectional study conducted in the Department of General Surgery at Agartala Government Medical College and Govind Ballabh Pant Hospital (AGMC & GBPH), Agartala, India, from May 2024 to November 2025.

Study population and selection criteria

The study population included all patients diagnosed and/or treated with gallbladder cancer at the study center between May 2024 and November 2025. All individuals with a confirmed diagnosis of gallbladder cancer during this period were eligible for inclusion, irrespective of stage (including locally advanced disease) or clinical presentation (including jaundice). Patients were excluded if they declined to participate, had a prior or concurrent diagnosis of any other malignancy, or had received any treatment for gallbladder cancer before enrolment.

Sample size and sampling

During the study period, a total of 123 patients were diagnosed with gallbladder cancer. Of these, 19 patients were excluded based on predefined exclusion criteria, resulting in a final sample of 104 patients for analysis. Non-probability sampling was employed for this study to include all eligible participants.

Data collection and data analysis

Data were collected from consenting patients with a confirmed diagnosis of gallbladder cancer attending the Department of Surgery, AGMC & GBPH, Agartala, India. Information was recorded using a predesigned and structured proforma capturing socio-demographic characteristics (age, gender, education, occupation, socioeconomic status, residence, dietary pattern, and addiction history), clinical variables (history of cholecystitis, comorbidities, and family history of cancer), and clinicopathological details (see Appendix 1). These comprised detailed clinical history, physical examination findings, laboratory investigations, including tumor markers (carbohydrate antigen 19-9 (CA19-9) and carcinoembryonic antigen (CEA)), radiological evaluations (abdominal USG and contrast-enhanced computed tomography (CECT) of the abdomen), image-guided fine-needle aspiration cytology (FNAC) reports, and tumor staging. Staging was performed according to the eighth edition of the American Joint Committee on Cancer (AJCC) TNM classification [18]. Socioeconomic status was assessed using the modified BG Prasad scale [19]. Data were analyzed using IBM SPSS Statistics for Windows, Version 26 (Released 2018; IBM Corp., Armonk, NY, USA) and presented as frequencies and percentages for categorical variables and as means with standard deviations for continuous variables. Chi-square or Fisher’s exact test was used to assess the association between categorical variables, and a p-value of <0.05 was considered statistically significant. 

Ethical considerations

The study was approved by the Institutional Ethics Committee for Clinical Studies of Agartala Government Medical College, Agartala, West Tripura, India (approval no. 6832). Written informed consent was obtained from all participants prior to enrolment. All diagnosed cases were subsequently referred to the Department of Oncology of AGMC & GBPH for further management.

## Results

The mean age of participants was 61.4 (±8.8) years, with more than half of the participants (n = 59, 56.7%) aged over 60 years. The cohort was predominantly female (n = 92, 88.5%). A substantial proportion of participants were homemakers (n = 73, 70.2%). Most participants had an education up to the primary level (n = 42, 40.4%), belonged to lower socioeconomic strata (n = 52, 50.0%), and were residents of rural areas (n = 78, 75%). A majority were non-vegetarian (n = 89, 85.6%) and reported betel nut chewing (n = 75, 72.1%). Detailed sociodemographic characteristics are depicted in Table 1. A previous history of cholecystitis was reported in 46 (44.2%) patients, while 37 (35.5%) participants had comorbidities, mainly hypertension (n = 23) and diabetes mellitus (n = 11); three patients had both hypertension and diabetes. A family history of cancer in a first-degree relative was noted in eight participants.

**Table 1:** Basic characteristics of participants (n = 104)

Variables	Sub-group	Frequency	Percentage
Age (in years)	<40	6	5.8%
	40-60	39	37.5%
	>60	59	56.7%
Gender	Male	12	11.5%
	Female	92	88.5%
Education	Illiterate	38	36.5%
	Primary school	42	40.4%
	High school	24	23.1%
Occupation	Unskilled	21	20.2%
	Service	10	9.6%
	Homemaker	73	70.2%
Socioeconomic status	Upper	13	12.5%
	Upper middle	9	8.7%
	Middle	14	13.5%
	Lower middle	16	15.4%
	Lower	52	50.0%
Residence	Urban	26	25.0%
	Rural	78	75.0%
Food habits	Vegetarian	15	14.4%
	Non-vegetarian	89	85.6%
Addiction	Smoking	11	10.6%
	Alcohol	15	14.4%
	Betel nuts	75	72.1%
	No addictions	3	2.9%

**Figure 1:**
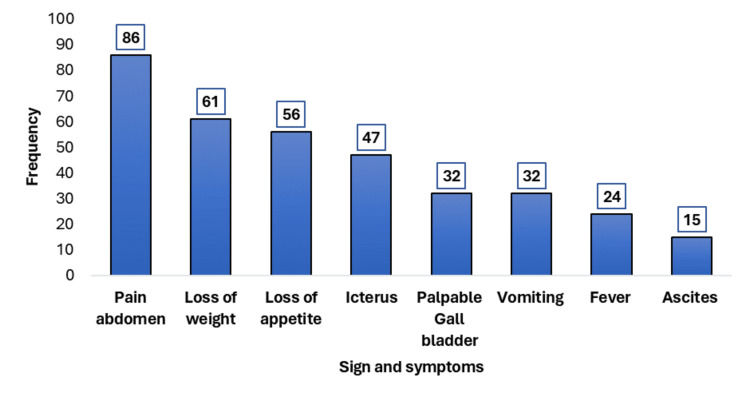
Distribution of presenting sign and symptoms (mutually inclusive)

Patients commonly presented with multiple symptoms. Abdominal pain was the most frequent presenting symptom (n = 86), followed by weight loss, loss of appetite, and vomiting. Icterus was the most common clinical sign (n = 47), followed by palpable gallbladder, fever, and ascites, which were also observed (Figure 1).

Laboratory findings at presentation are summarized in Table 2, showing elevated bilirubin levels in 62 (59.6%) patients, CEA in 69 (66.3%) patients, and raised CA19-9 in 77 (74%) patients.

**Table 2:** Details of laboratory findings at presentation (n = 104) TLC, total leukocyte count; CEA, carcinoembryonic antigen; CA19-9, carbohydrate antigen 19-9

Laboratory parameters	Category	Frequency	Percentage
Bilirubin (mg/dL)	Normal (<1.2)	42	40.4%
	High (>1.2)	62	59.6%
Hemoglobin (gm/dL)	Normal (>12)	24	23.1%
	Low (<12)	80	76.9%
TLC (cells/µL)	Normal (<11000)	72	69.2%
	High (>11000)	32	30.8%
Serum urea (mg/dL)	Normal (<20)	78	75.0%
	High (>20)	26	25.0%
Serum creatinine (mg/dL)	Normal (<1.5)	84	80.8%
	High (>1.5)	20	19.2%
CEA (ng/mL)	Normal (0-2.5)	35	33.7%
	High (>2.5)	69	66.3%
CA19-9 (U/mL)	Normal (0-39)	27	26.0%
	High (>39)	77	74.0%

Radiological evaluation revealed characteristic features of gallbladder malignancy on both USG and CECT. On USG of the whole abdomen, a mass arising from the gallbladder was the most frequent finding (n = 82, 78.8%), followed by gallbladder mass/lump with calculi (n = 78, 75.0%) and focal or diffuse asymmetrical wall thickening (n = 61, 58.7%). Loss of the interface between the gallbladder and liver was observed in 27 (26.0%) cases, while ascites (n = 17, 16.3%), intraluminal polypoidal masses (n = 15, 14.4%), and regional lymphadenopathy (n = 10, 9.6%) were less commonly detected. On CECT of the whole abdomen, gallbladder mass lesions were identified in 94 (90.4%) patients, with associated gallstones in 74 (71.2%). Asymmetrical gallbladder wall thickening was noted in 64 (61.5%) cases. Evidence of biliary obstruction, including common hepatic duct or common bile duct narrowing, was present in 37 (35.6%) cases. Regarding extension of the mass, hepatic infiltration involving segments IVB and/or V was observed in 55 (52.9%) cases, while involvement of adjacent organs such as the duodenum, colon, or stomach was seen in 26 (25.0%) cases. Vascular involvement, including right hepatic artery or portal vein encasement, was identified in 11 (10.6%) cases. Regional lymph-node metastases were detected in 67 (64.4%) patients, and distant metastatic disease in the form of lung or liver metastasis (separate from direct infiltration) and peritoneal metastasis or ascites was present in 28 (26.9%) cases (Table 3).

**Table 3:** Findings of radiological investigations (n = 104) USG, ultrasonography; CECT, contrast-enhanced computed tomography; GB, gallbladder; CHD, common hepatic duct; CBD, common bile duct; RHA, right hepatic artery; PV, portal vein

Radiological investigation	Findings	Frequency	Percentage
USG whole abdomen	Mass arising from the gallbladder	82	78.8%
	GB mass with calculi	78	75.0%
	Focal/Diffuse asymmetrical wall thickening	61	58.7%
	Loss of interface between GB & liver	27	26.0%
	Ascites	17	16.3%
	Intraluminal polypoidal mass	15	14.4%
	Regional lymphadenopathy	10	9.6%
CECT whole abdomen	Gallbladder mass lesion	94	90.4%
	Gallstones	74	71.2%
	GB asymmetrical wall thickening	64	61.5%
	Biliary obstruction/CHD or CBD narrowing	37	35.6%
	Hepatic infiltration (segments IVB/V)	55	52.9%
	Involvement of adjacent organs (duodenum, colon, stomach)	26	25.0%
	Vascular involvement (RHA/PV encasement)	11	10.6%
	Lymph-node metastases (regional)	67	64.4%
	Lung/liver peritoneal metastasis (separate from direct infiltration)/ascites	28	26.9%

**Figure 2:**
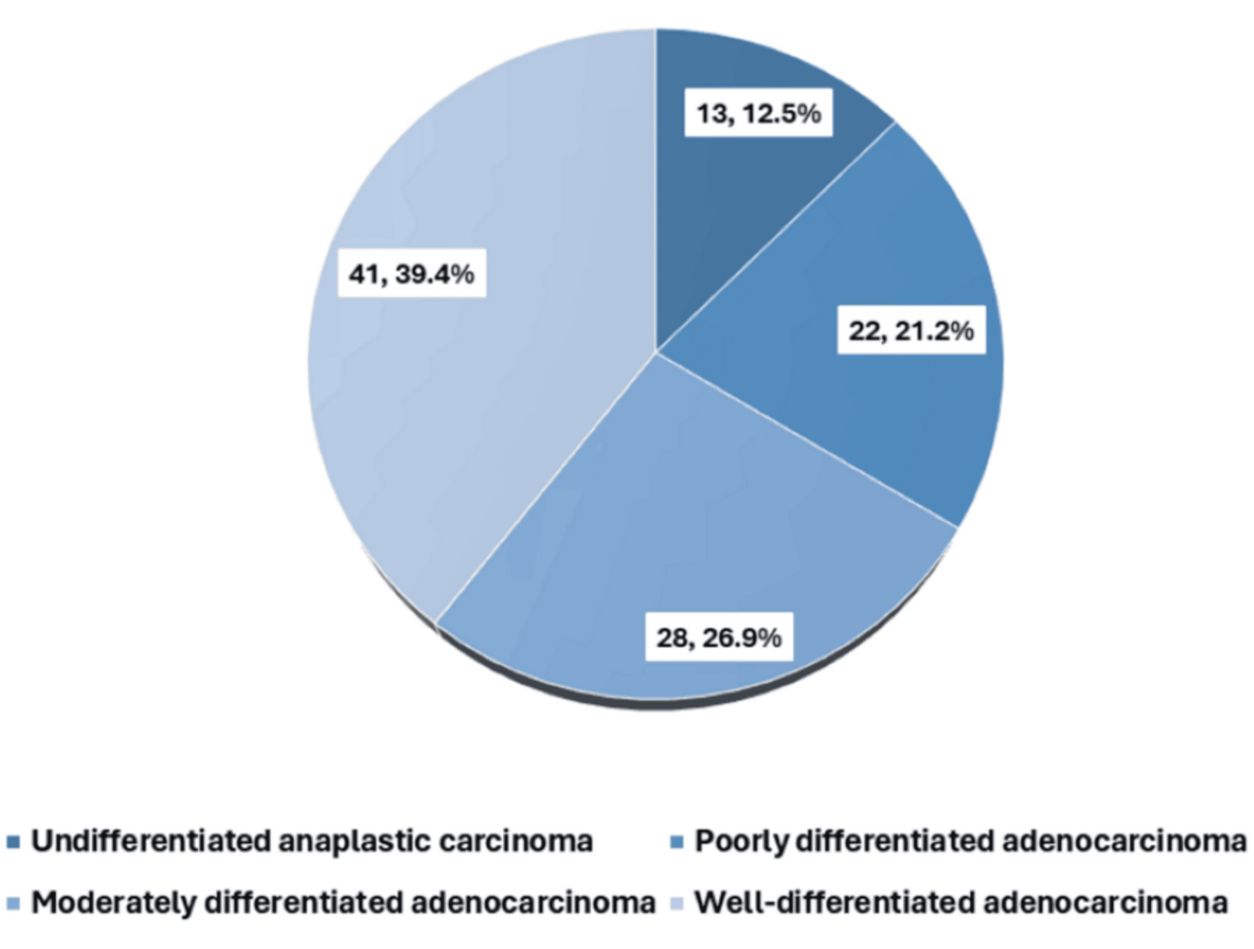
Histopathological distribution of the carcinoma (n = 104)

Image-guided FNAC revealed that well-differentiated adenocarcinoma was the most common subtype, accounting for 41 (39.4%) cases, followed by moderately differentiated adenocarcinoma and poorly differentiated adenocarcinoma. Undifferentiated anaplastic carcinoma was the least commonly observed histopathological variant, with 13 (12.5%) cases (Figure 2).

Table 4 depicts the details of Tumor, Node, Metastasis (TNM) staging. TNM staging demonstrated that the majority of patients presented with advanced primary tumors. T3 disease, characterized by serosal perforation or direct liver invasion or invasion of one other adjacent structure, such as the stomach, duodenum, colon, pancreas, or extrahepatic ducts, was the most common stage (n = 52, 50.0%), followed by T2 tumors (n = 29, 27.9%). With respect to regional lymph nodes, nearly half of the patients had N1 disease involving one to three regional nodes (n = 50, 48.1%), whereas 37 (35.6%) had no nodal involvement (N0), and 17 (16.3%) had N2 disease with involvement of four or more nodes. Distant metastasis (M0) was absent in the majority of patients (n = 76, 73.1%); however, 28 (26.9%) cases presented with metastatic disease (M1).

**Table 4:** Details of TNM staging (n = 104) TNM: tumor, node, metastasis

Details of TNM staging	Frequency	Percentage
T-Tumor		
T1 (tumor confined to lamina propria or muscle layer)	3	2.9%
T2 (invading peri-muscular connective tissue, no serosal breach)	29	27.9%
T3 (perforates serosa or direct liver invasion or one other adjacent structure like the stomach, duodenum, colon, pancreas, or extrahepatic ducts)	52	50.0%
T4 (invades main portal vein/hepatic artery or invades ≥2 adjacent organs)	20	19.2%
N-Regional Lymph Node		
N0 (no regional nodes)	37	35.6%
N1 (1-3 regional nodes)	50	48.1%
N2 (≥4 regional nodes)	17	16.3%
M-Metastasis		
M0 - no distant metastasis	76	73.1%
M1 - liver metastasis distinct from infiltration, peritoneal deposits, lung metastasis, and ascites	28	26.9%

**Figure 3:**
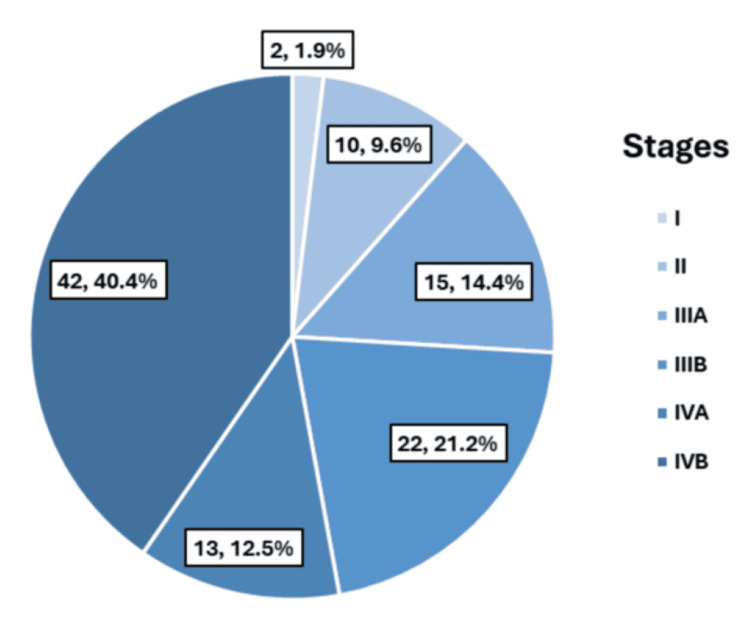
Distribution of staging of cancer according to AJCC classification (n = 104) AJCC, American Joint Committee on Cancer

Staging according to the AJCC classification demonstrated that most patients presented with advanced-stage disease. Stage IVB was the most common stage, accounting for 42 (40.4%) patients, followed by Stage IIIB in 22 (21.2%) patients and Stage IIIA in 15 (14.4%) patients (Figure 3).

Table 5 shows the association of histopathological type of carcinoma with selected sociodemographic and clinical variables. No statistically significant association was observed between histopathological type and age group, gender, socio-economic status, dietary habit, place of residence, addiction history, or history of cholecystitis (all p-values > 0.05).

**Table 5:** Association of histopathological type of carcinoma with sociodemographic and clinical variables (N = 104) Values in cells depict n (%); # denotes Chi-square, $ denotes Fisher’s Exact.

Variables	Anaplastic carcinoma (n = 13)	Adenocarcinoma (n = 91)	p-value
Age group (years)			
≤60	5 (10.9)	41 (89.1)	0.654^# ^(χ² = 0.200, df = 1)
>60	8 (13.8)	50 (86.2)	
Gender			
Female	12 (13.0)	80 (87.0)	1.000^$^
Male	1 (8.3)	11 (91.7)	
Socio-economic status			
Upper	2 (15.4)	11 (84.6)	0.918^$^
Middle (upper middle/middle/lower middle)	5 (12.8)	34 (87.2)	
Lower	6 (11.5)	46 (88.5)	
Dietary habit			
Non-vegetarian	10 (11.2)	79 (88.8)	0.395^$^
Vegetarian	3 (20.0)	12 (80.0)	
Place of residence			
Rural	11 (14.1)	67 (85.9)	0.510^$^
Urban	2 (7.7)	24 (92.3)	
Addiction history			
Absent	1 (33.3)	2 (66.7)	0.333^$^
Present	12 (11.9)	89 (88.1)	
History of cholecystitis			
Absent	7 (12.1)	51 (87.9)	0.881^# ^(χ² = 0.022, df = 1)
Present	6 (13.0)	40 (87.0)	

Table 6 shows that most patients presented with advanced disease (Stage III/IV). However, the clinical stage of carcinoma showed no statistically significant association with age group, gender, socio-economic status, dietary habit, place of residence, addiction history, or history of cholecystitis (all p-values > 0.05).

**Table 6:** Association of clinical stage of carcinoma with sociodemographic and clinical variables (N = 104) Values in cells depict n (%); # denotes Chi-square, $ denotes Fisher’s Exact.

Variables	Stage I/II (n = 12)	Stage III/IV (n = 92)	p-value
Age group (years)			
≤60	4 (8.7)	42 (91.3)	0.419^# ^(χ² = 0.653, df = 1)
>60	8 (13.8)	50 (86.2)	
Gender			
Female	11 (12.0)	81 (88.0)	1.000^$^
Male	1 (8.3)	11 (91.7)	
Socio-economic status			
Upper	2 (15.4)	11 (84.6)	0.754^$^
Middle (upper middle/middle/lower middle)	5 (12.8)	34 (87.2)	
Lower	5 (9.6)	47 (90.4)	
Dietary habit			
Non-vegetarian	10 (11.2)	79 (88.8)	0.683^$^
Vegetarian	2 (13.3)	13 (86.7)	
Place of residence			
Rural	10 (12.8)	68 (87.2)	0.726^$^
Urban	2 (7.7)	24 (92.3)	
Addiction history			
Absent	1 (33.3)	2 (66.7)	0.310^$^
Present	11 (10.9)	90 (89.1)	
History of cholecystitis			
Absent	9 (15.5)	49 (84.5)	0.154^# ^(χ² = 2.034, df = 1)
Present	3 (6.5)	43 (93.5)	

## Discussion

The present study highlights the clinicopathological and radiological profile of gallbladder cancer patients in a tertiary care center in Tripura, revealing significant findings consistent with and extending previous literature. A marked female predominance was observed, and the mean age at presentation was 61.4 years. These observations mirror established epidemiological trends, wherein gallbladder carcinoma is reported to occur two- to six-fold more frequently in women than in men, and predominantly affects individuals in the sixth to seventh decades of life [2,10,11]. A recent retrospective study by Priya et al. also reported a fourfold increased incidence of gallbladder carcinoma among females [20]. A study from the Sub‐Himalayan region of India by Gupta et al. also showed a three-fold greater incidence among females [21]. Hormonal influences, reproductive factors, and a higher prevalence of gallstone disease among women have been proposed as plausible biological explanations for this gender disparity in earlier studies [11]. A major proportion of patients belonged to lower socioeconomic strata and resided in rural areas. This pattern highlights the potential role of socioeconomic deprivation, limited access to healthcare services, delayed health-seeking behavior, and environmental exposures in shaping disease burden and stage at presentation. Similar associations between gallbladder carcinoma and lower socioeconomic status have been reported in the literature, suggesting that social determinants of health may significantly influence both risk and outcomes [11].

Lifestyle-related factors were also prominent in the present cohort. A high prevalence of betel nut chewing and consumption of a non-vegetarian diet was documented. These practices are culturally entrenched in many parts of Northeast India, and may contribute to chronic inflammation, exposure to carcinogens, and metabolic alterations implicated in gallbladder carcinogenesis [11,22-24]. Smoking and alcohol consumption were also reported among the study participants. Although causal relationships cannot be established from the present analysis, these findings are consistent with earlier reports, suggesting that lifestyle factors, including dietary patterns and substance use, may modify the risk of gallbladder cancer [25,26]. For instance, Aune et al., in a meta-analysis, demonstrated evidence of an increased risk of gallbladder disease associated with tobacco smoking, supporting the plausibility of smoking as a contributory risk factor in gallbladder carcinogenesis [27].

Clinically, abdominal pain was the most common presenting symptom, and a substantial proportion of patients presented with icterus, along with other clinical features such as a palpable abdominal mass and ascites, indicating biliary obstruction and peritoneal involvement. The predominance of abdominal pain is consistent with earlier studies that emphasize the nonspecific nature of early gallbladder cancer manifestations, which often contribute to delayed diagnosis [2,20,24]. Laboratory evaluation revealed elevated levels of tumor markers, with CA 19-9 increased in 77 (74%) patients, and CEA in 69 (66.3%) patients. This supports previous evidence suggesting their role as useful adjuncts in the diagnostic work-up and disease assessment of gallbladder carcinoma, although they lack specificity [2,7,8].

The detection rates of gallbladder mass lesions on USG (n = 82, 78.8%) and CECT (n = 94, 90.4%) in the present study demonstrate a pattern comparable to that reported in previous literature, reaffirming the diagnostic utility of these imaging modalities [7,8]. Furthermore, the high prevalence of regional lymphadenopathy (n = 67, 64.4%) and distant metastases (n = 28, 26.9%) observed on imaging reflects the advanced stage at presentation and the substantial metastatic burden associated with gallbladder carcinoma in this study population. These findings are indicative of the advanced stage at presentation, which is typical of this malignancy, and contribute to its poor overall prognosis [2,24,26]. Histopathological evaluation demonstrated a predominance of well-differentiated adenocarcinoma (n = 41, 39.4%). The additional presence of moderately differentiated and poorly differentiated adenocarcinoma, as well as undifferentiated anaplastic carcinoma, highlights the marked histological heterogeneity of this disease. These findings are consistent with global and Indian literature identifying adenocarcinoma as the predominant histological subtype of gallbladder cancer, supporting the glandular epithelial origin of most tumors. Variations in tumor differentiation are clinically relevant, as they have been associated with differences in biological behavior, aggressiveness, and patient outcomes in earlier studies [20,21,24,26]. Staging according to the TNM classification revealed that most patients presented with advanced disease. Half of the cohort had T3 tumors, indicating serosal perforation or direct hepatic invasion, while nearly half demonstrated regional lymph node involvement. Additionally, more than one-quarter of patients had evidence of distant metastasis at the time of diagnosis. This pattern of late-stage presentation is consistent with existing literature, which underscores the difficulty of early detection due to the largely asymptomatic nature of early disease and its clinical overlap with benign gallbladder conditions [2,10]. The predominance of Stage IVB disease in this cohort further reflects the advanced disease burden and parallels observations from other Indian and international studies, emphasizing the need for improved strategies for early diagnosis and timely intervention [21,24].

This study has a few strengths, including an integrated evaluation that combined clinicopathological, radiological, and histopathological findings, as well as its focus on the under-represented state of Tripura in Northeast India, thereby providing valuable region-specific epidemiological and clinical evidence. Nevertheless, certain limitations should be acknowledged. The single-center design limits the generalizability of the findings, while the use of non-probability sampling may have introduced selection bias, and the absence of longitudinal follow-up precluded assessment of prognosis and survival outcomes. Given the tertiary care setting, referral bias cannot be excluded. In addition, the absence of a control or comparison group limited the ability to evaluate risk factors or outcomes in a comparative manner. The absence of multivariable modeling further limits adjustment for confounders and restricts causal inference.

## Conclusions

This study provides a detailed clinicopathological and radiological characterization of gallbladder carcinoma in patients from a tertiary care center in Tripura, demonstrating a predominance of advanced-stage disease at presentation and a marked female preponderance. The high burden of regional lymph node involvement and distant metastasis indicates a propensity for early dissemination and advanced disease at presentation in this malignancy. By addressing an underrepresented geographic region, the findings contribute valuable region-specific evidence to the limited body of literature from Northeast India. While the prospective design, integrated clinicoradiological-histopathological assessment, and use of AJCC 8th edition staging strengthen the study, limitations related to its single-center nature, non-probability sampling, and absence of longitudinal follow-up and molecular profiling restrict generalizability and prognostic inference.

Future multicentric and longitudinal studies, incorporating molecular and genetic analyses, alongside targeted screening strategies and public health interventions addressing modifiable risk factors, are warranted to facilitate earlier diagnosis and improve outcomes in this high-risk population.

## Appendices

Appendix 1: Study proforma

Date of data collection:

Patient identification no.:

CR no.:

A) Basic Information

Age (in years):

Gender: Male/Female/Others

Address: Rural/Urban

Occupation:

Total family income (INR):

No of family members:

Socioeconomic status (BG Prasad):

B) Personal History

Diet: Vegetarian/Non-vegetarian

Alcohol: Yes/No

Smoking: Yes/No

Other substance use/addiction:

C) History of present illness:

D) Comorbidity: Yes/No, If Yes, Specify:

E) Past history of cholecystitis: Yes/No

F) Family h/o cancer: Yes/No

G) Laboratory Investigation

Bilirubin (mg/dL)

Hemoglobin (gm/dL)

TLC (cells/microliters)

Serum urea (mg/dL)

Serum creatinine (mg/dL)

CEA (ng/mL)

CA19.9 (u/mL)

H) Radiological Investigation

USG Whole Abdomen:

CECT Whole Abdomen:

USG/CT guided FNAC findings:

I) TNM & AJCC staging (write the TNM stage and tick the AJCC 8th stage)

Tumour (T):

Lymph Node (N):

Metastasis (M):

AJCC Stage: I/II/IIA/IIIB/IVA/IVB

J) Additional information (if any):
